# SAPHO syndrome with adrenal deficiency: a case report

**DOI:** 10.4076/1757-1626-2-6281

**Published:** 2009-08-14

**Authors:** Sibel Eyigör, Hale Karapolat, Hilal Adanur, Yeşim Kirazli

**Affiliations:** 1 Physical Therapy and Rehabilitation Department, Ege University Faculty of Medicine, 35100 Bornova, Izmir, Turkey

## Abstract

**Introduction:**

The SAPHO syndrome (synovitis, acne, pustulosis, hyperostosis, osteomyelitis) is a rare painful disorder, usually with a good long-term prognosis. Its etiology remains unclear, and various treatment regimens frequently fail to control the disease.

**Case presentation:**

A 46-year-old Caucasian female was referred for anterior chest wall and back pain. Physical examination was unremarkable except for skin lesions noted on soles of both feet, extremities and the face. A thoracic magnetic resonance imaging study demonstrated a lesion characterized with bone marrow edema and proliferation of soft tissue in the sternum. A brain MRI was requested secondary to the elevated prolactin level which was compatible with empty sella syndrome.

**Conclusion:**

The case presented here has the unique feature of adrenal deficiency presenting alongside the SAPHO syndrome and is presented as the first case reported. This syndrome could become complicated with different organ system involvement other than bone and skin. There is a need further studies that will explore the weak relationship between SAPHO syndrome and adrenal deficiency.

## Introduction

The SAPHO syndrome (synovitis, acne, pustulosis, hyperostosis, osteomyelitis) is a rare chronic painful disorder first described by Chamot *et al. *in 1987 [1]. Even though it could be encountered at any age, the most frequent presentation is during childhood or middle age and the course is characterized by relapses and remissions. The most frequent and the most problematic complaint is bone pain. Skin lesion such as pustular psoriasis, acne, and suppurative hydraadenitis could also be present [2,3]. The treatment is difficult and often inadequate, despite good prognosis, involvement of multiple organ systems could complicate the disease course [4]. The SAPHO syndrome has been linked with bacteriological, immunological, and genetic mechanisms; however, the exact etiology still remains a mystery [2,5]. The case presented here has the unique feature of adrenal deficiency presenting alongside the SAPHO syndrome and is presented as the first case reported.

## Case presentation

A 46-year-old Caucasian female patient from Turkey presented with complaints of three-month old back and chest wall pain. The prescibed nonsteroidal antiinflammatory drugs (NSAID) had alleviated the symptoms; however, skin lesions especially on the soles of the feet erupted during the same period. The patient complained of intermittent fatigue. Past medical history was significant for epilepsy and four sinus surgeries. The family history was noncontributory.

**Figure 1:**
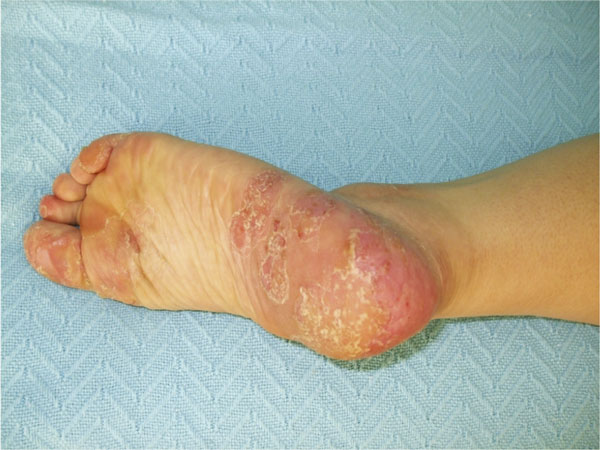
**Skin lesions were seen on soles of both feet**.

Physical examination was unremarkable except for skin lesions noted on soles of both feet, extremities and the face. The lesions were interpreted as pustular psoriasis by dermatology (Figure 1).

Laboratory investigation obtained for differential diagnosis included complete blood count, routine biochemistry, 24-hour urine for protein, protein and immunoglobulin electrophoresis, Rheumatoid factor, anti-nuclear antibodies, anti-DNA, complements, immunoglobulins, tumor markers, hepatitis serologies, group agglutinations tests and were all within normal limits. There was no Bence-Jones proteinuria and the patient was HLAB27 negative. Among the hormone tests (FSH, LH, DHEAS, estradiol, progesteron, insulin) requested, prolactin levels were found to be elevated (50.48 ng/ml, normal range: 4.8-23.3). ACTH basale level was <10 (normal range: 10-46) and cortisol level was 11.26 (normal range: 5-25 μg/dl). Cortisol response to insulin was normal. A throat culture obtained secondary to postnazal discharge only revealed normal flora. The cultures of the bacteriological specimes obtained from the plantar lesions remained without growth. The chest X-ray was normal. An abdominal ultrasound demonstrated grade 2 hepatosteatosis. A bone densitometry revealed osteopenia of the hip.

Cervical X-rays demonstrated only straightening of the cervical lordosis and nonspecific degenerative changes. A lumbosacral X-ray obtained showed degenerative changes of the facets. The sacroiliac, feet and heel graphies were all within normal limits. Bone scan revealed focal area of increased activity in mid-sternal region. A thoracic magnetic resonance imaging (MRI) study demonstrated a lesion characterized with bone marrow edema and proliferation of soft tissue in the upper 1/3 of the sternum. Clinical and radiological findings led to the diagnosis of the SAPHO syndrome. A brain MRI was requested secondary to the elevated prolactin level which was compatible with empty sella syndrome with bowl-like widening of the sella, decreased gland height and spread inside the sella. The patient was additionally diagnosed with "adrenal deficiency" under stress. The patient was prescribed difluortolone 2 valerate- chlorquinaldole 10 mg creme for skin lesions and a NSAID (Diclofenac 75 mg BID) for pain. Despite improvement of pain control, the skin lesions continued to demonstrate waxing-and-waning course (Figure 1). For adrenal deficiency, the patient was started on hyrocortisone 15 mg p.o. daily and Prednol 20 mg IV p.r.n. as stress dose. ACTH and cortisol levels were to be monitored at monthly intervals.

After six months, the patient had less chest wall pain with NSAID. The skin lesions continued to demonstrate waxing-and-waning course. There are no signs or laboratory values of suspected systemic involvement.

## Discussion

An example of the rarely occuring SAPHO syndrome, with early diagnosis and the first reported concomitant occurance of adrenal deficiency is presented here.

Several bacteriological, immunological, and genetic mechanisms have been proposed as the etiology and thought to be involved in the pathogenesis of the SAPHO syndrome; however, to this day, the exact etiology remains unclear [4,5]. The most frequently recovered pathogen from the bone lesions seen in this syndrome is Propionebaterium acnes; however, in many cases as in ours the pathogenic organism can never be identified [6]. There are ongoing debates regarding possible links to HLA profile and ankylosing spondylitis in the etiology. While thirteen percent of all the patients are found to be HLA B27 positive, most patients are negative as in the case presented here [7]. However, some authors maintain that the SAPHO syndrome can be regarded as a seronegative spondyloarthropathy due to the presence of high incidence of sacroiliitis, enthesopathy, axial involvement, inflammatory bowel disease and HLAB27 carriage and the absence of rheumatoid factor [6]. Maugars et al. have reported that 7 patients out of 21 have met the European classification criteria for spondyloarthropathy [8]. However, our patient does not have findings compatible with seronegative spondyloarthropathy. Even though there is no validated diagnostic criteria, a patient has to fulfill at least one criterion described by Benhamou *et al. *[9] for diagnosis (i). severe acne accompanying joint lesions (ii). severe palmoplantar pustulosis and joint involvement (iii). osteohypertrophy of the sternoclavicular joint, spine or the extremities (iv). chronic recurrent multiple osteomyelitis. In the absence of skin lesions, the last two criteria could be adequate for diagnosis. However, this investigator has been criticized for not including bone lesions in the diagnostic criteria [5]. According to records of 120 patients with long-term follow-up, 84% of the patients present with skin lesions similar to our patient [10]. While the chest wall is frequently involved in this syndrome (clavicula, sternum, sternoclavicular joint), involvement of spine, ribs, tarsal region, mandibula, and long bones have also been reported [10,11]. The most prevalent complaint in the majority of cases is chest pain [12]. Our patient did not have complaints other than back and chest wall pain and skin lesions. With these complaints and the presence of pustular psoriasis and sternal involvement, the patient was thought to meet the diagnostic criteria for the SAPHO syndrome.

Laboratory results are nonspecific in the SAPHO syndrome and the requested work-up is important for differential diagnosis [3,11]. Our patient did not have any abnormal laboratory findings except the eleveated prolactin level. The role of prolactin in the interrelation between the endocrine system and the immune system has a biphasic character. Generally lesswell known is that prolactin may also play a role in the activity of autoimmune diseases such as systemic lupus erythematosus and rheumatoid arthritis. Further studies should be carried out on this subject to understand the exact mechanism. The hormonal tests obtained were helpful in the diagnosis of adrenal deficiency which might have gone unrecognized otherwise.

Physical examination and clinical assessment are important for reaching the right diagnosis. In patients who only have atypical findings, a bone biopsy taken from the inflamed bones could become necessary for definitive diagnosis [5,6,11].

The radiological findings in the SAPHO syndrome, while reported on quite extensively, are nonspecific [13]. The most prominent feature is the presence of hyperosteosis and osteitis. Bone scan findings are nonspecific and bone scan should be performed in the asymptomatic phase or when direct radiological examinations are normal [3]. Computerized tomography (CT) could clearly show hyperostotic and lytic lesion and magnetic resonance imaging (MRI) can be used in the definitive localization of active inflammatory changes [6,13]. MRI is important in the follow-up of the disease and is quite sensitive. In our patient, other than sternal involvement, there was no bone involvement found in the imaging studies.

Different disease groups should be entertained in the differential diagnosis of the SAPHO syndrome. The radiographical changes in the long bones could be confused with infectious osteomyelitis and bone tumors (such as Ewing sarcoma). In cases with severe multiple destructive lesions, metastatic bone involvement must be ruled out. There is also reports of autoimmune disease such as rheumatoid arthritis presenting simultaneously with the SAPHO syndrome [3].

Treatment is not necessary in the SAPHO syndrome. NSAIDs recommended for pain are effective and used frequently [3,14]. Our patient had significant pain relief after symptomatic treatment with NSAIDs. In cases with prominent bone involvement, steroids, colchicin, doxycyclin, salazopyrine, interferone and bisphosphonates have been reported to be effective [3]. The anti-osteoclastic and antiinflammatory effects of bisphosphonates are beneficial in treatment [2]. The early use of bisphosphonates in the early stages of the disease has been reported to be beneficial for the management of the bone lesions, however, caution must be exerted regarding the adverse effects of the medication on kidneys and the quality of the bone [6]. In cases with more prominent skin lesions rather than bone involvement and when the pathogenic organism can be identified, antibiotics are preferred over biphosphonate treatment secondary to lower cost and widespread availibility [6]. Positive experiences with anti-TNF agents are also reported. However, these medications, despite being effective on osteoarticular complaints, could lead to exacerbation of the skin lesions [14]. The steroids added to the therapeutic regimen of NSAIDs for the treatment of the adrenal insufficency in our patient are thought to potentially be effective on the bone lesions as well.

## Conclusion

The case presented herein is the first reported case of the quite rare SAPHO syndrome with adrenal deficiency. We have no hypothesis to better understand this association. There is a need further studies that will explore the weak relationship between SAPHO syndrome and adrenal deficiency. This syndrome could become complicated with different organ system involvement other than bone and skin. We believe that systematic evaluation with review of all organ systems during work-up would be beneficial in determining early diagnostic and therapeutic strategies.

## Abbreviations

ACTH: adrenocorticotropic hormone; CT: computerized tomography; DHEAS: dehydroepiandrosterone; FSH: follicle stimulating hormone; LH: luteinizing hormone; MRI: magnetic resonance imaging; NSAID: nonsteroidal antiinflammatory drugs; SAPHO: synovitis, acne, pustulosis, hyperostosis, osteomyelitis

## Competing interests

The authors declare that they have no competing interests.

## Consent

Written informed consent was obtained from the patient for publication of this case report and accompanying images. A copy of the written consent is available for review by the Editor-in-Chief of this journal.

## Authors' contributions

SE was the major contributor in writing the manuscript, analyzed and interpreted the patient data. HK involved in collection of data, analyzed and interpreted the patient data. HA performed the general examination, analyzed and interpreted the patient data. YK supervised the group, analyzed and interpreted the patient data. All authors read and approved the final manuscript.
